# Suicide Risk in Personality Disorders: A Systematic Review

**DOI:** 10.1007/s11920-023-01440-w

**Published:** 2023-08-29

**Authors:** Heather McClelland, Seonaid Cleare, Rory C. O’Connor

**Affiliations:** Suicidal Behaviour Research Laboratory, Institute of Health & Wellbeing, University of Glasgow, Glasgow, Scotland

**Keywords:** Suicide, Personality Disorders, Review, Psychosocial factors, Interventions

## Abstract

**Purpose of review:**

This systematic review aimed to distil recent literature investigating psychosocial factors which may account for the association between personality disorder (PD) and suicide attempt or suicide death.

**Recent findings:**

Suicide risk is particularly elevated in people with PD compared to those with no, or many other, mental health diagnoses. Despite this, suicide prevention strategies for PD populations have not progressed markedly in recent years. It is critical, therefore, to identify additional factors associated with suicide in PD populations.

**Summary:**

Of the 34 studies included in this review, most identified a relationship between personality disorder and suicide attempt and/or death. Historical interpersonal factors (e.g., childhood trauma), drug and alcohol use, and ideation-to-enaction factors were commonly associated with suicide-related outcomes. Interventions that provide interpersonal support may reduce suicide attempts. Limitations of the review include the heterogeneity of studies and small sample sizes.

**Supplementary Information:**

The online version contains supplementary material available at 10.1007/s11920-023-01440-w.

## Introduction

Suicide is a global health concern with approximately 703,000 people dying by suicide each year [1]. Although research has identified myriad factors which may increase an individual’s risk of suicide (e.g., depression, lack of social support, social disadvantage), our ability to predict suicide remains limited [2]. The most consistent predictors of a future suicide attempt are a history of self-injurious behaviour including suicide attempt [3] or non-suicidal self-injury (NSSI) [4], with literature also indicating that repeated suicidal behaviour becomes more medically severe over time [3–5].

It is well established that suicidal behaviour is more common in those with mental health diagnosis than in those without. Indeed, some estimates suggest that as many as 90% of those who die by suicide have a diagnosable psychiatric disorder at the time of their death [6]. A recent meta-analysis which focused on repetition of suicide attempts found that the presence of any mental health diagnosis was associated with approximately double the increased risk of further suicide attempt. What is more, the risk of repeated suicide attempt was particularly elevated in those diagnosed with a psychotic or personality disorder [5].

Personality disorders (PD) are common, chronic mental health conditions. A recent meta-analysis of 46 studies from 21 countries found rates of PD in community-based research samples of 7.8% [7]. In clinical samples, estimates range from 30% for inpatient populations to 67% in outpatient samples [8, 9]. Globally, Borderline PD is estimated to occur in 1.6% of the general population and 20% of the psychiatric inpatient population [10]. Cross-national prevalence rates for other PDs in the general population are harder to establish. In some cases, prevalence rates are reported for PD clusters, as identifying specific PDs can be challenging [11]. For instance, to receive a diagnosis of antisocial personality disorder, a diagnosis of conduct disorder prior to the age of 15 is required [12].

Symptoms of PD often present first in adolescence or early adulthood, causing clinically significant distress or impairment over a long period of time [13]. Personality disorders are generally characterised by distressing patterns of emotions, thoughts and behaviours, and impaired interpersonal functioning. There are three main clusters of personality disorders; Cluster A encompasses patterns of unusual thinking or behaviours, Cluster B is characterised by unstable emotions, and problems with impulse control, and Cluster C features very anxious emotions, thoughts and behaviours (DSM-5) [14].

Within personality disorders, Cluster B conditions, such as borderline (BPD) and antisocial personality disorders (ASPD), are particularly associated with suicide risk and self-injurious behaviours. Indeed, enduring suicidal ideation and self-injurious behaviours are key features of BPD with an estimated 65–80% of patients with BPD engaging in NSSI [13, 15]. One clinical study of 394 patients with BPD recorded suicide attempts in 75% of their sample, and as many as 9% will die by suicide [15, 16]. It is estimated that around 5% of those with ASPD will die by suicide; however, these patients are more likely to be treated in forensic settings rather than clinical services and feature less in research around PD and suicidality [13].

Currently, it remains difficult to identify specific individuals within these high-risk groups who are more likely to take their own lives than others who will not [2, 17, 18]. The most recent version of the International Classification of Diseases (ICD-11) applies a dimensional approach to PD features and focuses on assessing the level of impairment across domains of functioning such as self and interpersonal areas and the distress associated with the impairment [19].

This echoes a similar movement in the field of suicidality where the recognition of a need to look beyond psychiatric diagnoses and symptoms has been a key driver in the development of recent models of suicidal behaviour such as the biopsychosocial model of suicide risk [20]. Models such as the biopsychosocial model consider the development of suicide risk in the context of a complex interplay of biological, psychological, social, and environmental factors across the lifespan.

Although biological determinants of suicide cannot easily be modified, psychological factors are more malleable. Turecki et al. (2019) highlight that the need for further research into modifiable, psychological factors associated with suicide, which may shed more light on how suicidal behaviour manifests and therein, offer more avenues of exploration for suicide prevention strategies [20].

Another important development in the field of suicide research in recent years has been the application of ideation-to-enaction frameworks. These frameworks posit that the factors associated with the emergence of suicidal ideation are different from those that govern engaging in suicidal behaviour [21]. One such model which addresses this distinction is the integrated motivational-volitional (IMV) model of suicidal behaviour. The IMV model incorporates components from other areas of health, psychology, and suicide research to map out the final common pathway to the emergence of suicidal ideation and suicidal behaviour [22–24].

The IMV model is a tripartite diathesis-stress model (pre-motivational, motivational, and volitional phases) which highlights that to understand the role of psychological factors, suicide risk must be considered within an individual’s biopsychosocial context. It posits that against the backdrop of existing vulnerability and triggering events, factors such as reduced social support, perceived burdensomeness, and thwarted belongingness (so-called motivational moderators) increase the risk of being trapped by mental pain and suicidal ideation emerging. In turn, the likelihood that suicidal ideation will be acted upon and that someone engages in self-harming behaviours is increased by the presence of a group of factors called volitional moderators. These factors include impulsivity, having ready access to the means of suicide, and fearlessness of dying/death (O’Connor & Kirtley, 2018). Importantly, these volitional factors are potentially modifiable, and therefore, may be useful targets for psychosocial interventions in the treatment of at-risk individuals.

The differentiation between factors associated with ideation and suicidal behaviour is crucial in the context of personality disorders, particularly BPD, as suicidal thoughts and behaviours are relatively common [25]. Consequently, in this review, we aim to map out recent literature focusing on psychosocial factors associated with suicidal behaviour in populations with PD.

### Current Study

The aim of this review is to summarise recent published literature exploring the association between psychosocial factors in individuals with a diagnosis of personality disorder and suicidal behaviour.

## Methods

A systematic search of literature was conducted across five databases (CINHAL, Medline, PsychArticles, PsychInfo, and Web of Science) on 27th January 2023. Subject heading terms relating to self-harm, suicide, and personality disorders were developed using Boolean search terms (e.g., AND, OR; see Appendix 1 for a full list of search terms). Included papers were articles where (i) suicide or a confirmed suicide attempt was the outcome, (ii) the target population had a confirmed diagnosis of a personality disorder, (iii) they were written in English, (iv) they were published in a peer-reviewed journal, and (v) they were published since 2020. Titles and abstracts, then full-text papers, were screened for eligibility with inter-rater checks completed by two of the study authors. Discrepancies were resolved through discussion (see Appendix 2 for Prisma statement and inter-rater reliability details). Categorisation of the psychosocial factors for the sub-analysis (see the “Psychosocial Determinants” section) was conducted independently by two of the review authors.

## Results

Thirty-four papers were eligible for this review, these studies are summarised in Table 1. In total, data from 199,320 participants were included in this review. 32.6% of the sample was male, and on average, participants were 30.9 years old (sd. 10.1; based on 23 studies).

**Table 1:** Study summaries

**Author**	**Design, site, country**	**Whole sample demographics**	**PD type**	**Outcome**	**Result**
Alberdi-Páramo et al. (2020) [50]	Cross-sectional, Personality Disorders Day Unit of the Hospital, Spain	*N* = 109 Male: 33% Age: 18–56	Borderline	Suicide attempt	• Symptoms of anxiety^3^ and depression^3^ were significantly greater in suicide attempt groups than non-suicide attempt
Alberdi-Páramo et al. (2021) [53]	Cross-sectional, Personality Disorders Day Unit of the Hospital, Spain	*N* = 104 Male: 27.6% Age: 18–56	Borderline	Suicide attempt	• Suicide attempt method^4^ involved a combination of methods for BPD populations • BPD participants with a NSSI history^4^ were significantly more likely to make a suicide attempt
Allen et al. (2022) [44]	Prospective (30 years), Inpatient, outpatient, and community referral sources, USA	*N* = 458 Male: 23% Age: 18–50 (28.59 ± 7.53)	Borderline	Suicide attempt	• The association between interpersonal dysfunction^2^ and suicide attempt in BPD populations was significant. Suicidal ideation fully accounted for this association
Aouidad et al. (2020) [26]	Prospective (four years) Inpatient unit following suicide attempt, France	*N* = 320 Male: 17.0% Age: 11.5–17.7 (14.7 ± 1.29)	Borderline	Suicide attempt	• Insecure attachment style^2^, negative life events in the past year^2^, anxiety^3^, OCD^3^, depression^3^ and substance dependence^3^ were all significant associated with PD and suicide attempt
Bozzatello et al. (2022) [45]	Prospective (1 year), University, Outpatients, Italy	*N* = 1019 Male: 36.9% Age: 51.01 ± 15.32	Borderline	Suicide attempt	• The association between BPD and suicide attempt was moderated by community functioning^2^
Broadbear et al. (2020) [46]	Retrospective (psychological post- mortem), Suicide reported to the Coroners Court of Victoria (CCOV), Australia	*N* = 2870 Male: 49.7% Age: 18–64	Borderline	Suicide death	• Contextual (e.g., physical abuse)^2^, romantic (e.g., domestic violence) ^2^ or historical stress (e.g., school bullying) ^2^, substance use^3^, suicide from a friend or family member^4^ were significantly associated with suicide in BPD populations
Chanen et al. (2022) [54]	Cross-sectional, Monitoring Outcomes of BPD in Youth (MOBY) dataset, Australia	*N* = 139 Male: 19.4% Age: 15–25 (19.1 ± 2.8)	Borderline	Suicide attempt	• History of suicide attempt in the last 12 months^4^ was not significantly associated with current suicide attempt in BPD populations
Cunningham et al. (2021) [27]	Cross-sectional, Veterans, USA	*N* = 124 Male: 24.2% Age: 23–77 (48.7 ± 13.0)	Borderline	Suicide attempt	• Within a multiple variable hierarchical analysis, BPD was not significantly associated with suicide attempt^1^
Ducasse et al. (2020) [47]	Cross-sectional, Hospital inpatients, France	*N* = 92 Male: 22.0% Age: 37 ± 12.6)	Borderline	Suicide attempt	• Emotional dysregulation^2^, impulsiveness^2^, childhood trauma (not physical or emotional neglect) ^2^, preoccupied attachment pattern (but not other attachment styles) ^2^, shame proneness^2^ were all significantly associated with suicide attempt in BPD populations
Edwards et al. (2022) [28]	Cross-sectional, Veterans, USA	*N* = 282 Male: 73% Age: 20–57 (40.67 ± 10.75)	Borderline	Suicide attempt	• BPD was significantly associated with suicide attempt^1^
Flynn et al. (2020) [51]	Retrospective (psychological post-mortem), NCISH, UK	*N* = 1601 Gender: NA Age: NA	Any	Suicide death	Method of hanging/ jumping^4^ was not significantly associated with suicide attempt. Substance use^3^, violence^2^ and self harm^2^ were significantly associated with suicide death
Fruhbauerova et al. (2021) [29]	Prospective (1 year), Active military, USA	*N* = 148 Male: 85.9% Age: 26.8 ± 5.9	Borderline	Suicide attempt	• BPD was not significantly associated with suicide attempt^1^
Gec et al. (2021) [55]	Intervention (10-weeks) Community and internal service hospital referrals, Australia	*N* = 42 Male: 9.1% Age: 20–53 (33.3 ± 8.6)	Borderline	Suicide attempt	• 12 of the 13 participants to report a suicide attempt within 12 months prior to baseline, did not report suicide attempt during the 10-week intervention^5^
Girardi et al. (2022) [30]	Psychological autopsy, Discharged from community mental health, Italy	*N* = 54,350 Male: 41.9% Age: 48 (Median)	Borderline	Suicide death	• BPD was significantly associated with suicide death^1^ • All non-borderline PD diagnoses were significantly associated with suicide attempt^1^
Grilo and Udo (2021) [31]	Cross-sectional, General population (NESARC), USA	*N* = 20,442 Male: 56.3% Age: 45.6 ± 17.5	Borderline	Suicide attempt	• Lifetime history^4^ and 12-month history^4^ of suicide attempt were significantly associated with suicide attempt
Høye et al. (2021) [32]	Cross-section In- and outpatient specialist health care services, Norway	*N* = 32,885 Male: 40.0% Age: 20–79 (37.3 ± 12.0)	Any	Suicide death	• PD participants with substance use disorder^3^ were significantly more likely to die by suicide than PD participants with an alternative comorbidity or no dual diagnosis
Hui and Hilton (2022) [33]	Retrospective, High security psychiatric hospital, Canada	*N* = 486 Male: n/a Age: 35.86 ± 11.78	Antisocial	Suicide attempt	• Suicide attempt group were significantly more likely to have a diagnosis of Antisocial PD than the non-suicide attempt group^1^
Jiang et al. (2021) [34]	Psychological autopsy; Danish Cause of Death Registry 1995–2015, Denmark	*N* = 14,103 Male: N/A Age: 40 (SD = 23)	Any	Suicide death	• Any PD was not significantly associated with suicide attempt^1^ • Any PD was significantly associated with suicide death^1^
Kuehn et al. (2020) [48•]	Intervention (2-year RCT), local healthcare services, USA	*N* = 97 Male: 0% Age: 18–60	Borderline	Suicide attempt	• Avoidant coping^2^, relationship satisfaction^2^ and emotion regulation^3^ were not significantly associated with suicide attempt • Emotion regulation strategies^3^ and problem focused coping^2^ were significantly associated with suicide attempt
Lee et al. (2020) [49•]	Cross-sectional, Korean university hospital inpatient, South Korea	*N* = 125 Male = 43.7% Age: 18–65 (28.39 ± 9.18)	Borderline	Suicide attempt	• 21 clinical symptoms^3^ were explored of which 14(hysteria symptoms, anxiety symptoms, obsessive–compulsive, phobic anxiety symptoms, depression symptoms, clinical comorbidities, general symptom index, positive symptom distress level, positive symptom total, hypochondriasis, hypomania symptoms, psychopathic deviate symptoms, psychoticism symptoms, somatization) were not significantly associated with suicide attempt. However, seven clinical symptoms were (masculinity-femininity symptoms, over- and under-reporting of PD symptoms, paranoia symptoms, paranoid ideation symptoms, psychasthenia symptoms, schizophrenia symptoms) were significantly associated with suicide attempt^3^ • Of four interpersonal factors explored^2^, interpersonal sensitivity and social introversion were not significantly associated with suicide attempt in BPD populations whereas defence mechanisms and hostility were significantly associated with suicide death in this population sample
Martin and Del-Monte (2022) [56]	Prospective (intervention study), University hospital and a psychiatric clinic, France	*N* = 56 Male: 0% Age: 49.16 ± 9.19	Borderline	Suicide attempt	• Suicide attempts were significantly reduced for the treatment as usual group when compared to the ‘Cognitive and Behavioural Skills Related to Emotional Observation and Regulation’ treatment group^5^
McMain et al. (2018) [35]	Intervention (RCT, 24 months), Suicidal or self-harm patients, Canada	*N* = 240 Male: 21% Age: 18–60 (28.27 ± 8.62)	Borderline	Suicide attempt	• When compared to DBT 6-month treatment group, suicide attempts were significantly reduced in the DBT 12-month treatment group at 6-month and 12-month intervention follow-up but not at 24-months ^5^
Papadopoulou et al. (2020) [36]	Cross-sectional, Psychiatric inpatient referrals following emergency presentation of suicide attempt, Greece	*N* = 231 Male: 32.9% Age: 21–80	Any	Suicide attempt	• Compared to bipolar disorder, participants with PD were significantly less likely to have a repeated attempt history^1^
Pompili et al. (2022) [37]	Cross-sectional, Psychiatric patients, Italy	*N* = 2137 Male: 38.6% Age: 42.87 ± 14.98	Any	Suicide attempt	• Patients who attempted suicide were significantly more likely to have a borderline, or alternative personality disorder, when compared to patients who did not attempt suicide^1^
Reutfors et al. (2021) [38]	Case control study; Diagnosed with depression in specialised care, Sweden	*N* = 15,631 Male: 42% Age: 18–69	Any	Suicide attempt	• Participants with a diagnosis of any PD were significantly more likely to report a history of suicide attempts when compared to depression populations^1^
Schmeck et al. (2022) [57] Basel, Heidelberg	Intervention (Non- RCT), Child and Adolescent Psychiatric Hospital, Chile	*N* = 60 Male: 7% Age: 15.78 ± 1.35	Borderline	Suicide attempt	• Suicide attempts were not significantly reduced for either Dialectical Behaviour Treatment for Adolescent or Adolescent Identity Treatment intervention groups when compared to baseline^5^ • No significant differences in suicide attempt prevalence was observed between Dialectical Behaviour Treatment for Adolescent and Adolescent Identity Treatment groups at follow-up^5^
Sekowski et al. (2022) [39]	Cross-sectional, Inpatient public psychiatric hospital, Poland	*N* = 389 Male: 43.45% Age: 12–17 (14.76 ± 1.47)	Borderline	Suicide attempt	• BPD participants reported a significantly greater number of suicide attempts than those with sub-threshold BPD or without BPD^1^
Söderholm et al. (2020) [40]	Retrospective, Psychiatric secondary care outpatients, Finland	*N* = 124 Male: 32.1% Age: 18–50	Borderline	Suicide attempt	• Lifetime history of suicide attempt was significantly greater in BPD patients than bipolar or major depressive disorder populations^3^
Sommer et al. (2021) [52]	Cross-sectional; General population; National Epidemiologic Survey on Alcohol and Related Conditions, USA	*N* = 36,309 Male: NA Age: NA	Borderline	Suicide attempt	• Depersonalisation^3^ and dissociation^3^, but not derealisation^3^, was significantly associated with suicide attempt in participants with BPD when compared to participants with posttraumatic stress disorder
Sumlin et al. (2020) [41]	Cross-sectional; Inpatient treatment programme, USA	*N* = 162 Male: 33% Age: 12–17 (15.33 ± 1.42)	Borderline	Suicide attempt	• Compared to psychiatric patients with no PD diagnoses, BPD patients were more likely to have a lifetime history of attempted suicide^1^ • Having a BPD diagnosis was not significantly associated with suicide death^1^
Walton et al. (2020) [58]	Intervention (14 months), Specialist outpatient service for BPD and/or eating disorders, Australia	*N* = 162 Male: 23% Age: 26.6 ± 7.8	Borderline	Suicide attempt	• Participants receiving DBT were significantly more likely to report suicide attempts at baseline compared to participants receiving Conversational Model therapy^5^ • Suicide attempt did not significantly differ between participants receiving DBT or Conversational Model at 7- month follow-up^5^ • Participants receiving DBT were significantly less likely to report suicide attempts at 14-month follow-up when compared to participants receiving Conversational Model therapy^5^
Wolf et al. (2022) [42]	Prospective (10 weeks), Psychiatric hospital ward Germany	*N* = 170 Male: 32.6% Age: NA	Borderline	Suicide attempt	• Prevalence of suicide attempts did not significantly differ between Borderline PD and persistent depressive disorder participants’ groups^4^
Yen et al. (2021) [43]	Prospective (10 years); Inpatient, partial, and outpatient treatment settings, USA	*N* = 701 Male: 36% Age: 33	Borderline, Obsessive compulsive	Suicide attempt	• Obsessive compulsive and Borderline, but not Antisocial PD, were significantly associated with suicide attempt^1^

*BPD* borderline personality disorder, *DBT* dialectical behavioural therapy, *N* total number of participants, *N/A* not available, *NCISH* National Confidential Inquiry into Suicide and Safety in Mental Health, *NESARC* National Epidemiologic Survey on Alcohol and Related Conditions, *NSSI* non-suicidal self-harm, *PD* personality disorder, *RCT* randomised controlled trial

Sub-groups: ^1^overall association between PD and suicide-related outcomes; ^2^interpersonal factors; ^3^affective states and clinical symptoms; ^4^ideation to enact ion factors; ^5^intervention

**Fig. 1:**
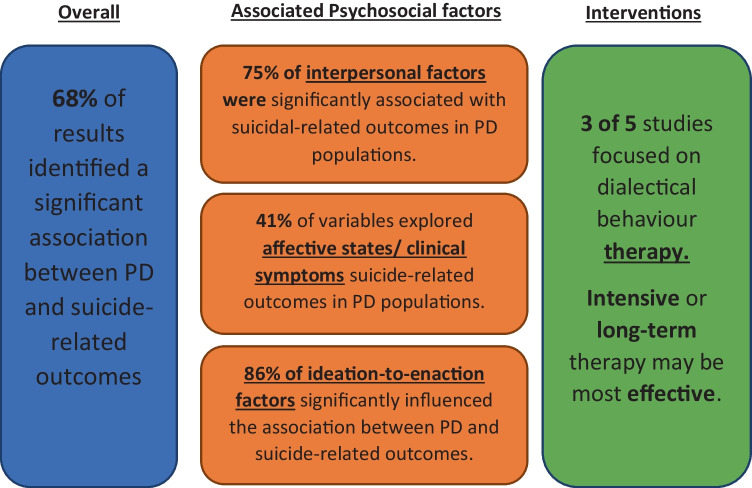
Association between personality disorder and suicide-related outcomes

Of the 34 papers, 19 (25 results) investigated the overall association between PD and suicide attempt-related outcomes, whereas 20 (74 results) explored psychosocial factors which mayinfluence the association between PD and suicide-related outcomes (four papers investigated both overall association and possible influencing factors). Consequently, the results are presented in three sections (see Fig. 1):

1. Overall association between PD and suicide-related outcomes
2. Psychological determinants of suicide-related outcomes in PD populations
3. Interventions

### Overall Association Between Personality Disorder and Suicide-Related Outcomes

Across 24 results (18 studies), 17 results indicated that any form of PD was significantly associated with suicide-related outcomes [26–43]. 21 results (18 studies) measured PD in relation to suicide attempts, of which 14 reported a significant association. There was no discernible trend to distinguish between studies which did or did not report a significant association. Four studies measured suicide death as an outcome, with three reporting a significant association with PD [30, 32, 34, 41]. Only Sumlin et al. (2020) reported no association between PD and suicide death; however, the sample size of this study was considerably smaller (*N* = 162) than that of the other three studies (range: from 14,103 to 54,350 participants).

Across three studies, two found the prevalence of suicide attempt or death to be lower in people with PD than in studies of individuals with diagnoses of depression [36, 38]. Whereas, Söderholm et al. (2020) found that prevalence of suicide attempts was greater in PD populations than populations with depression [40].

### Psychosocial Determinants

Thirteen studies explored psychosocial factors which may explain the association between PD and suicide-related outcomes, yielding 63 independent results. For the purposes of this review, these psychosocial factors were grouped into three subheadings (interpersonal factors, affective states and ideation-to-enaction factors; see Table 1).

#### Interpersonal Factors

Seven studies (19 results) explored the role of interpersonal factors in the association between PD and suicide-related outcomes [26, 44–47, 48•, 49•]. All seven studies exclusively recruited participants with BPD. The interpersonal factors included historical events (e.g., childhood trauma, attachment style, school bullying) as well as current, contextual interpersonal factors (e.g., romantic status or satisfaction, shame proneness) and coping styles. Fourteen of the 19 results indicated a significant association between BPD and suicide-related outcomes. The five results (three studies) where interpersonal factors were not found to be associated with suicide-related outcomes in PD populations explored current internal cognitions (specifically relationship satisfaction, interpersonal sensitivity, social introversion, and avoidant coping) or negative life events in the past year [26, 48•, 49•]. These non-significant results were identified in studies in which suicide attempt was the outcome, and typically they recruited smaller sample sizes (*N* ≤ 125 participants).

#### Affective States and Clinical Symptoms

Nine studies (37 results across 30 variables) explored affective states and mental health diagnoses [26, 44, 46, 47, 48•, 49•, 50–52]. Three variables (anxiety symptoms, depression symptoms, emotion regulation) were measured in three studies each, with mixed results in relation to suicide attempt [26, 44, 46, 47, 48•, 49•, 50–52]. Conversely, three studies (four results) measured substance use (including drugs and alcohol) and each found that it explained, in part, the relationship between any PD and suicide death [26, 46, 51].

Of the remaining 21 variables measured (four studies, 25 results), 13 did not indicate a significant association with suicide-related outcomes in PD populations [44, 47, 49•, 51, 52]. Six of these 13 non-significant results included some aspect of emotional detachment (e.g., derealisation, hypomania, somatization), whereas results that reported a significant association between PD and suicide-related outcomes, were more likely to relate to cognitions (e.g., paranoia, defence mechanisms). In addition, studies with larger sample sizes (*N* > 400 participants) were also more likely to report a significant association between affective states or symptoms and suicide-related outcomes [44, 52].

#### Ideation-to-Enaction Factors

Six studies (nine results) explored three factors relating to ideation-to-enaction of suicide [26, 46, 47, 51, 53, 54]. Of these three factors, all indicated a significant association with suicide attempt or death in PD populations. The only exception to this was Chanen et al. (2022) where a history of suicide attempt in the last 12 months was not significantly associated with current suicide attempt within a BPD-only sample [54]. Additionally, Flynn et al. (2020) found that compared to other mental health diagnoses, self-poisoning with drugs, but not hanging or jumping, was more prevalent in those with a diagnosis of PD [51].

### Interventions

The leading psychosocial intervention for PD is dialectical behavioural therapy (DBT), with a well-established evidence-base. However, DBT is not effective for everyone, and in this review, four different psychosocial approaches were explored.

Five studies (eight results) investigated psychological interventions, with only suicide attempt, not suicide death, measured as an outcome variable [35, 55–58]. Furthermore, these studies only recruited from BPD populations. Three studies investigated DBT; however, the comparison treatment groups differed between these studies (see Table 1) [35, 57, 58]. Martin and Del-Monte (2022) investigated Cognitive Behavioral Therapy for emotional regulation (CBT-E) and was the only study to have treatment as usual (TAU) as the comparison group [56]. Schmeck et al. (2022) compared DBT to Adolescent Identity Treatment (AIT), whereas Gec et al. (2021) explored an Intensive Group Program (IGP) comprising of elements of DBT, CBT, and acceptance and commitment therapy approaches with no comparison group [55, 57].

Martin and Del-Monte (2022) found no significant reduction in suicide attempts in the CBT-E participant group; however, there was a significant reduction in suicide attempts in the TAU group [56]. The treatment for the TAU group included support groups, weekly occupational therapy groups and regular meetings with a psychiatrist or a health professional. Schmeck et al. (2022) found no significant difference in the prevalence of suicide attempts between AIT and DBT intervention groups at baseline or at follow-up [57]. Additionally, compared to baseline, neither intervention was associated with significant reductions in suicide attempt at follow-up. Walton compared suicide attempts in a group receiving DBT to those receiving Conversational Model therapy [58]. Although results did not differ significantly between these therapeutic approaches, both interventions led to a significant reduction in suicide attempts at 14-month follow-up. Similarly, McMain et al. (2018) found that when compared to participants with BPD who received 6-months of DBT, participants with BPD receiving 12-months of DBT treatment, reported significantly fewer suicide attempts at 6- and 12-month follow-up, [35]. Although Gec et al. (2021) did not have a comparison group, following 10 weeks of IGP (delivered in 4-h sessions, 2 days per week) only one of the 13 participants with a baseline history of suicide attempt in the last 12 months, made another attempt during the intervention [55].

## Discussion

This systematic review summarises the research literature exploring suicide attempt and/or death in populations with a diagnosis of personality disorder (PD), published within the last 3 years. The results indicate that both suicide attempt and suicide death are sadly evident in those with PD, with ideation-to-enaction factors and interpersonal factors (especially historical interpersonal factors, e.g., childhood trauma), explaining, in part, the association between PD and suicide risk. Additionally, substance use was found to be consistently associated with suicide-related outcomes in PD populations, whereas the role of affective states (e.g., depression, anxiety) was less conclusive. This review also revealed that most interventions for PD were focused around dialectical behaviour therapy (DBT). Studies which showed a reduction in suicide attempt over time were studies with longer treatment engagement or more direct engagement with treatment services [35, 56]. Pioneering research conducted outside this paper’s review period also indicates that cognitive analytic therapy (CAT) may also be effective for the treatment of PD. Despite this, no such studies were identified in this review.

Although many studies summarised in this review found significant associations between PD and suicide-related outcomes, these associations were not consistent, and the psychosocial factors identified herein did not fully explain the relationship. This illustrates the complexity of the pathways to suicidal behaviours and that although a mental health diagnosis is associated with an increased propensity for suicidal behaviour, it does not make it inevitable, with other, contextual factors playing significant roles [17, 18]. The research included in this review indicates the potential role of psychosocial factors such as interpersonal problem-solving, but more research is needed to understand how, and when, such factors are associated with suicidal ideation and suicide attempts in these groups.

In addition, most of the research included herein focused on BPD, which, given its associated increased risk of suicidal behaviour, is understandable. However, focusing on understanding the role of psychosocial factors within the context of different personality disorders and how these are differentially associated with suicide risk may also further our understanding of the mechanisms through which suicide risk might increase. Applying frameworks such as the IMV model to build theoretically guided, testable hypotheses of the pathways to suicide may advance our understanding of suicide risk in these populations [17]. For instance, according to the IMV model, defeat and entrapment are key components in the emergence of suicidal ideation; however, the importance of these factors in the early identification of suicide risk in those with personality disorders is unclear [17].

The recent introduction in the ICD-11 of assessing the level of impairment across domains of functioning opens up other avenues to understanding the complex relationship between PD and suicide risk [19]. This perspective may further our understanding of the interactions between factors, while highlighting that the importance of each factor will vary across individuals, making the lived experience of suicidal thoughts and behaviours subjective and unique; this is something that should be reflected upon in future research.

There is also a need to explore how different PD presentations are experienced by individuals, particularly how they affect their mental health, their coping response, and their suicide risk. Qualitative as well as quantitative research is required to address these gaps in knowledge. For the most part, the research herein has focused on risk factors; therefore, it may be helpful for future research to explore factors which may offer individuals some protection against suicide risk.

McMain et al. (2018) were the only study included in this review to explore the longer-term (2 years) effects of therapeutic interventions [35]. The evidence of this review indicates that individuals with personality disorder may benefit from on-going support, with effects of psychological treatment after discharge potentially being short-lived.

### Limitations

This review must be considered within the context of its limitations. First, there was considerable heterogeneity between the studies, with few studies including the same variables or therapeutic approaches; so it is difficult to determine consistency. Second, this review did not include the role of biology or pharmacology in the association between personality disorder and suicide-related outcomes. Also, this was a very focused review, primarily looking at studies published in the last 3 years; consequently, there is a risk that some important research may have been missed. It is noteworthy that no qualitative studies were included in this review.

Additionally, within the current review, most papers reported a significant association between PD and suicide-related outcomes. Given that papers are more likely to be published if they report a significant result, exploring publication bias would be advantageous. Lastly, the sample size of the included studies may have influenced the detection of significant associations. Of the included studies, those with a sample size of > 400 participants were more likely to report a significant association with suicide-related outcomes than studies with a smaller sample size. Despite the frequency of suicide attempts being higher in PD populations compared to non-PD populations, as the relative prevalence of suicide attempt and of death remains low it is more difficult to detect significant effects in the studies with smaller sample sizes.

### Conclusions

This review distils findings published within the last 3 years which explored diagnosed personality disorder in relation to suicide attempt and/ or suicide death. Most studies found that this association remains significant and may be linked to substance use and historical interpersonal factors such as attachment style and childhood trauma. In this review, there is evidence to suggest that psychosocial treatments may be protective of suicide attempt in BPD populations; however, their protective effectives may be influenced by duration and intensity of these treatment programmes. This review also highlights the need for more in-depth research to better understand the factors, mechanisms, and circumstances, associated with increased risk of suicide in individuals with personality disorders. Studies which did not report significant results typically had a smaller sample size. Due to the wide variety of variables investigated in the included studies, future work would benefit from replicating the existing associations as well as conducting meta-analytic analyses.

## Supplementary Information

Below is the link to the electronic supplementary material.

Supplementary file1 (PDF 223 KB)
