# Supplementary material for: Suicide Risk in Personality Disorders: A Systematic Review
Source: Curr Psychiatry Rep. 2023 Aug 29;25(9):405–17. doi: 10.1007/s11920-023-01440-w (PMC10506938; doi:10.1007/s11920-023-01440-w)
Supplement: Supplementary file 1 — Supplementary file1 (PDF 223 KB) [file 11920_2023_1440_MOESM1_ESM.pdf]

Appendix 1 Search terms

Database(s)	Search (s)	Terms	Hits
CINHAL, Medline, PsychArticles, PsychInfo	S1	MH "Suicide") OR (MH "Self-Injurious Behavior") OR (MH "Suicide, Completed") OR (MH "Suicide, Attempted") OR (MH "Self Mutilation") **OR** (MH "Self-Injurious Behavior") OR (MH "Suicide") OR (MH "Self Mutilation") **OR** MM "Nonsuicidal Self-Injury" OR MM "Self-Inflicted Wounds" OR (MH "Wounds, Stab") OR (MM "Self Mutilation") OR (MM "Self-Injurious Behavior") OR "self-harm" OR "self-poison*' OR self-injur* OR attempted suicide or suicidal attempt or suicide attempt OR suicidal thoughts OR suicidal ideation **OR** MM "Nonsuicidal Self-Injury" OR MM "Self-Inflicted Wounds" OR (MH "Wounds, Stab") OR (MM "Self Mutilation") OR (MM "Self-Injurious Behavior") OR "self-harm" OR "self-poison*' OR self-injur* OR attempted suicide or suicidal attempt or suicide attempt	
	S2	(MH "Antisocial Personality Disorder") OR (MH "Compulsive Personality Disorder") OR (MH "Schizoid Personality Disorder") OR (MH "Paranoid Personality Disorder") OR (MH "Histrionic Personality Disorder") OR (MH "Schizotypal Personality Disorder") OR (MH "Borderline Personality Disorder") OR (MH "Dependent Personality Disorder") OR (MH "Passive-Aggressive Personality Disorder") OR (MH "Personality Disorders") OR (MH "Dissociative Identity Disorder") **OR** "Antisocial Personality Disorder" OR DE "Avoidant Personality Disorder" OR DE "Borderline Personality Disorder" OR DE "Dependent Personality Disorder" OR DE "Histrionic Personality Disorder" OR DE "Narcissistic Personality Disorder" OR DE "Obsessive Compulsive Personality Disorder" OR DE "Paranoid Personality Disorder" OR DE "Passive Aggressive Personality Disorder" OR DE "Sadomasochistic Personality" OR DE "Schizoid Personality Disorder" OR DE "Schizotypal Personality Disorder" **OR** (MH "Antisocial Personality Disorder") OR (MH "Histrionic Personality Disorder") OR (MH "Passive-Aggressive Personality Disorder") OR (MH "Dependent Personality Disorder") OR (MH "Multiple-Personality Disorder") OR (MH "Borderline Personality Disorder") OR (MH "Avoidant Personality Disorder") OR (MH "Narcissistic Personality Disorder") OR (MH "Schizotypal Personality Disorder") OR (MH "Compulsive Personality Disorder") OR (MH "Personality Disorders") **OR** "emotionally unstable personality disorder"	
	S3	S1 'AND' S2	**840**
Web of Knowledge		(TS= ("personality disorder")) AND TS=(suicid* OR self-harm OR self harm OR self-injurious OR self injurious OR Self mutilation)	
		1. 5 years	**594**

		2. Doc type<br>a. Article<br>b. Other<br>c. Unspecified<br>d. Early access<br>3. English<br>4. Mesh headings:<br>a. Borderline personality disorder, self-injurious behaviour, suicide attempted, suicide, personality disorders	

Text in bold denotes separate search groups.
Prior to extraction from online databases, papers were limited to the following factors in order; last five years (since 2018), academic article, written in English.

Appendix 2 Prisma statement

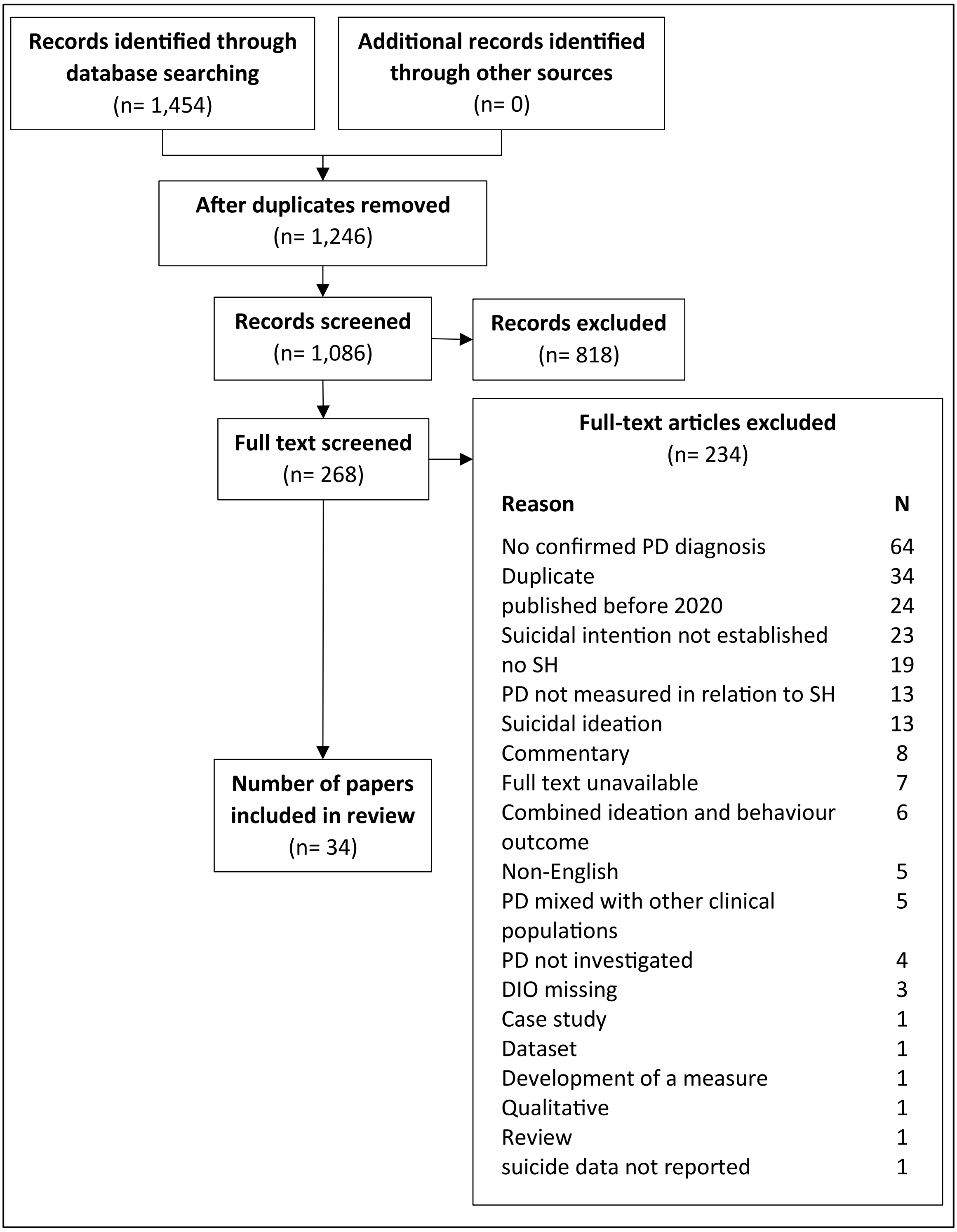

Criteria

Inclusion criteria: i) suicide, or confirmed suicide attempt was the outcome variable, ii) predictor variables were psychosocial in nature, iii) the target population had a confirm diagnosis of a personality disorder, iv) written in English, v) a peer-reviewed journal article, v) published since 2020.

Exclusion criteria: i) conference abstracts or posters, ii) non-journal articles (e.g., book chapters, reviews, editorials, case studies, commentaries, conference proceedings, protocol papers, editorial), iii) measured euthanasia or assisted suicide, iv) data collected using second-hand accounts, v) brain imaging or pharmaceutical studies, vi) homicide-suicide was the outcome, vii) studies assessing the validity of questionnaires, and viii) studies which did not explore suicide death or suicide attempt as a standalone outcome variable.

Inter-rater reliability

- Title and abstract (n= 60) screening inter-rater reliability was 87.8% with 100% concordance following discussion.
- Inter-rater of full-text papers (n= 18) was 94.4% with 100% concordance after discussion.
- The concordance rate for grouping of variables into categories was 91.8%, rising to 100% following discussion.
